# LASTR is a novel prognostic biomarker and predicts response to cancer immunotherapy in gastric cancer

**DOI:** 10.3389/fonc.2022.1020255

**Published:** 2022-09-29

**Authors:** Jun-Yan Liu, Jing Yao, Jia-Jia Liu, Tao He, Fang-Jie Wang, Tian-Yu Xie, Jian-Xin Cui, Xiao-Dong Yang

**Affiliations:** ^1^ Department of General Surgery, The First Medical Center, Chinese People's Liberation Army (PLA) General Hospital, Beijing, China; ^2^ Department of General Surgery and Center of Minimal Invasive Gastrointestinal Surgery, Southwest Hospital, Third Military Medical University (Army Medical University), Chongqing, China; ^3^ State Key Laboratory of Trauma, Burns and Combined Injury, Army Medical Center (Daping Hospital), Army Medical University, Chongqing, China

**Keywords:** lncRNA LASTR, ferroptosis, prognosis, biomarker, gastric cancer

## Abstract

Gastric cancer (GC), a malignant tumor of digestive tract, is characterized by a high death rate. Thus, it is of particular importance to clarify the mechanisms of GC and gain new molecular targets for the sake of preventing and treating GC. It was reported that long non-coding RNAs (IncRNAs) are prognostic factors to cancer. Ferroptosis refers to a process of programmed cell death dependent on iron. This study sets out to investigate the expression and function of ferroptosis-related lncRNA (FRlncRNA) in GC. TCGA datasets offered RNA-seq data for 375 GC patients and clinical data for 443 GC patients. Based on Pearson’s correlation analysis, we studied their expression and identified the FRlncRNAs. Differentially expressed prognosis related to FRlncRNA were determined with the help of the Wilcoxon test and univariate Cox regression analysis. To evaluate the accuracy of the prognostic capacity, researchers used the Kaplan-Meier technique, as well as univariate and multivariate Cox regression and receiver operating characteristic (ROC) curve studies. We also carried out the real-time PCR and CCK8 assays to examine the expression and function of FRlncRNA. In this study, we identified 50 ferroptosis-related DEGs which were involved in tumor progression. In addition, we identified 33 survival-related FRlncRNAs. Among them, lncRNA associated with SART3 regulation of splicing(LASTR) was confirmed to be highly expressed in GC specimens compared to non-tumor specimens in this cohort. Survival assays illuminated that the high LASTR expression predicted a shorter overall survival and progression-free survival of GC patients. Based on multivariate Cox regression analyses, it was confirmed that the GC had a worse chance of surviving the disease overall if their tumors expressed LASTR, which was an independent prognostic indication. Then, Loss-of-function tests showed that knocking down LASTR had a significant effect on reducing the proliferation of GC cells. Finally, we found that the expression of LASTR was negatively associated with CD8 T cells, T cells, Th17 cells, and T helper cells. Overall, our findings identified a novel survival-related FRlncRNA, LASTR which possibly can serve as a novel prognostic biomarker predicting response to cancer immunotherapy and therapeutic target for GC patients.

## Introduction

Gastric cancer (GC) is the third leading cause of cancer-related death (1). Globally, it is the fifth most common cancer with up to 1 million new gastric cancer cases and 783, 000 deaths projected in 2018 (2). While the number of inpatient admissions for GC has been dropping in the last few decades, the healthcare burden and expenditure it brought about experienced a tremendous growth (3, 4). There are many factors accounting for the occurrence of GC, including alcoholic abuse, irregular dieting, incorrect dieting habit and gene heredity (5, 6). Whether the perioperative chemotherapy and radiotherapy are implemented or not, the prognosis of GC patients are still poor (7, 8). An integrated understanding of the molecular mechanism of GC is conducive to develop efficient therapeutic methods and to ameliorate the clinical results of GC patients (9, 10). At present, surgical resection and chemotherapy are the first choices of GC treatments, since they can considerably avoid the recurrence and metastasis of GC. Consequently, to find out a sensitive diagnostic approach for the early gastric caner and facilitate prognosis, it is necessary to elucidate the molecular biological mechanism of this cancer.

Growing evidences from high-throughput sequencing have demonstrated that <2% of genes have protein-coding capacity, while >75% of gene transcripts are non-coding RNAs (11, 12). Therefore, concentrating on protein-coding genes may be ineffective to explore the mechanisms associated with tumorigenesis. A collection of non-coding transcripts that are more than 200 nucleotides long are known as long non-coding RNAs (lncRNAs) (13). While they are extremely similar in genetic organization, they play a variety of roles at the cellular level, such as regulating transcription and translation, altering gene expression and regulating the expression of chromosomally neighboring genes (14, 15). According to the findings of a number of studies, lncRNAs are at the center of a wide variety of physiological and pathological processes, including the progression of the cell cycle, the occurrence of apoptosis during the process of cellular differentiations, and immune function (16, 17). They are essential components in the processes of post-transcriptional control, transcriptional repression and chromatin remodeling (18, 19). In addition, more and more studies have reported that some functional lncRNAs possibly can serve as novel diagnostic and prognostic biomarkers for tumor patients (20, 21). However, to date, only few studies have documented lncRNAs in GC and the underlying mechanisms remain largely unknown.

Ferroptosis refers to a form of iron-dependent programmed cell death, featuring lipid peroxidation (22). With the development of study, ferroptosis is engaged in various crucial biological processes, such as cancer, ischemia-reperfusion injury, and neurodegenerative maladies (23). Ferroptosis is a crucial regulatory mechanism for tumor growth, and it is central to the chemoradiotherapy and immunotherapy treatment of malignancies, according to several studies that were conducted not too long ago (24, 25). Thus, these therapeutic approaches, when paired with drugs that target iron death signals, assist to increase their overall effectiveness against tumors. In gastric cancer, it was shown that the lncRNA known as PVT1 was elevated and had a significant relationship to both high microvessel density and a bad prognosis. Based on the gain- and loss-of PVT1 expression, PVT1 was found to be able to evidently trigger angiogenesis within tumors, apart from enhancing tumor growth by means of modulating the STAT3/VEGFA axis *in vitro* and *in vivo*. It should be noted that the carcinogenesis of lncRNA PVT1 is linked to ferroptosis (26). Up till now, it is not yet known in its entirety how ferroptosis-related lncRNAs contribute to the advancement of GC, and it is imperative that their vital significances in GC treatment and prognosis be further defined in the future.

This study identified 33 survival-related Ferroptosis-Related lncRNAs(FRlncRNA). Among them, our attention focused on lncRNA associated with SART3 regulation of splicing(LASTR) which was highly expressed in our cohort and TCGA datasets. Moreover, we analyzed the prognostic value of LASTR expression among patient with GC and the potential function *in vitro* experiments. These findings may provide a new a prognostic marker and therapeutic target for patients with GC.

## Materials and methods

### Clinic Samples

This study was composed of 11 GC specimens and the matched 11 para-tumorous specimens. Three pathologists were responsible for the confirmation of the histologic diagnosis. Patients participating in the study received surgery at The First Medical Center, Chinese PLA General Hospital, and the fresh tissues were snap-frozen in liquid nitrogen and preserved at −80°C for the next studies. We also gained the written informed consent of all patients and the approval of the Ethics Committee of The First Medical Center, Chinese PLA General Hospital.

### Cell culture and transfection

The Type Culture Collection of the Chinese Academy of Medical Science supplied the control human GES-1 gastric mucosa epithelial cells as well as the four GC cell lines (HGC-27, BGC-823, AGS and MGC-803). In a humidified incubator with 5 percent CO_2_, RPMI-1640 medium (Hyclone, Massachusetts, USA) with 10 percent fetal bovine serum (Hyclone) and penicillin/streptomycin was used to cultivate the cells. Oligonucleotides, including negative control siRNA and LASTR siRNA were synthesized by GenePharma (Shanghai, China). Lipofectamine 3000 was employed to transfect GC cells for *in vitro* assays. 48 hours after this transfection, cells were collected for the downstream analyses.

### RNA isolation and RT-PCR

TRIzol (Invitrogen; Thermo Fisher Scientific, Inc.) was applied to extract total RNA from GC samples and cells, and then the total RNA was reverse-transcribed into cDNA using SuperScript Reverse Transcriptase III (Invitrogen, China) in accordance with the instructions provided by the manufacturer. The following parameters were utilized for quantitative PCR reactions that were carried out using an ABI 7500 PCR System with the use of SYBR-Green Supermix (Applied Biosystems; Thermo Fisher Scientific, Inc.): Initial denaturation at 95 degrees Celsius for thirty seconds, followed by forty cycles of denaturation at 95 degrees Celsius for five seconds, annealing at 60 degrees Celsius for thirty seconds, and then a study of the melting curve. Fluorescent signals detection was carried out after every cycle. Using the 2^−ΔΔCq^ methods, relative mRNA expression levels were determined after being normalized to either GAPDH. The primer sequences were presented in **Table 1**.

**Table 1:** The primer sequences included in this study.

Name	primer sequences (5’–3’)
LASTR: forward	AGTGGGTGAAGTCCTGGTT
LASTR: reverse	GGCTGAAGGGTTTAGATG
LINC01711: forward	AGGTCAGGCCATACCCA
LINC01711: reverse	CCAGCCATCAGGTTCTGT
LINC01094: forward	TGTAAAACGACGGCCAGT
LINC01094: reverse	CAGGAAACAGCTATGACC
LINC01614: forward	CAACCAAGAGCGAAGCCAAG
LINC01614: reverse	CGCCCCAAAACAACTGAGTC
GAPDH: forward	GGAGCGAGATCCCTCCAAAAT
GAPDH: reverse	GGCTGTTGTCATACTTCTCATGG

### CCK-8 assay

Cells that were in the log phase were collected 24 hours after the transfection and planted into a 96-well plate at a density of 5 x 10^3^ cells per well, with 100 μL of media present in each well. At certain time intervals (24 h, 48 h, and 72 h), 10 μL of CCK-8 was added, and the mixture was then incubated with the cells for an additional 2 hours. In the end, the optical density(OD) values of the cells were examined at a wavelength of 450 nm using a MultiskanTM FC microplate reader manufactured in the United States by Thermo ScientificTM. The abscissa represented the passage of time, whereas the ordinate was denoted by OD value.

### Data collection

The Genomic Data Commons Data Portal was used to obtain the RNA-sequencing and clinical information for the GC samples that were part of the TCGA (GDC1). Additionally, we downloaded a “HTSeq-FPKM” workflow type of transcriptome profiling for the TCGA-STAD project. This workflow type of transcriptome profiling included the gene expression profiles of 375 cancer tissue samples and 32 normal tissue samples. The “bcr xml” file type was used to obtain the clinical information for all 443 GC tissues that came from the TCGA-STAD.

### Ferroptosis-associated lncRNAs

FerrDb is the first manually curated resource for regulators and markers of ferroptosis, and it was launched in December 2019; we were able to obtain 259 gene sets linked with ferroptosis from this database (22). Our group applied the limma package in R for the identification of the ferroptosis-associated lncRNAs that were found based on the correlation analysis between ferroptosis-related genes and IncRNA expressions in the GC samples. This analysis was based on the fact that there was a positive correlation between these two factors. After calculating the Pearson correlation coefficients, the threshold was determined to be a correlation coefficient of > 0.4 and a probability value of < 0.001.

### Functional enrichment analysis of the DEGs

The DEGs between GC specimens and non-tumor specimens were filtered in light of specific criteria (|log2FC| ≥ 2 and FDR < 0.05). On the basis of these DEGs, GO and KEGG analyses were accomplished with the help of the “clusterProfiler” package (27).

### Identification of the survival-related ferroptosis-related LncRNAs

Ferroptosis-related lncRNAs related to survival were assessed by means of univariate Cox regression analysis.

### Analysis of the relationships Between LASTR and prognosis

The survival date from the samples of TCGA was also available. Overall survival (OS) and progression-free survival(PFS) were thought to be the indicators for the exploration of relevancy between LASTR expression and patient prognosis. With respect to survival analyses, the Kaplan-Meier method and log-rank test were adopted in each cancer type. The survival curves were drawn using R packages “survival” and “survminer”. Besides, the R packages “forestplot” was used to figure out the relationship between LASTR expression and survival in pan-cancer.

### Statistical analysis

R statistical software version 4.0.4 and strawberry-perl-5.32.0.1 were employed to conduct all the statistical analyses. The two independent groups were compared using the Student’s t-test. We also conducted the univariate Cox regression analysis and multivariate Cox regression analysis to determine OS’s independent prognostic factors. Moreover, we carried out time-dependent ROC curve analysis to assess the predictive accuracy of the prognostic model for OS. Statistical significance was defined as p value <0.05 with all p values two-tailed.

## Results

### Enrichment analysis of ferroptosis-related DEGs

**Figure 1:**
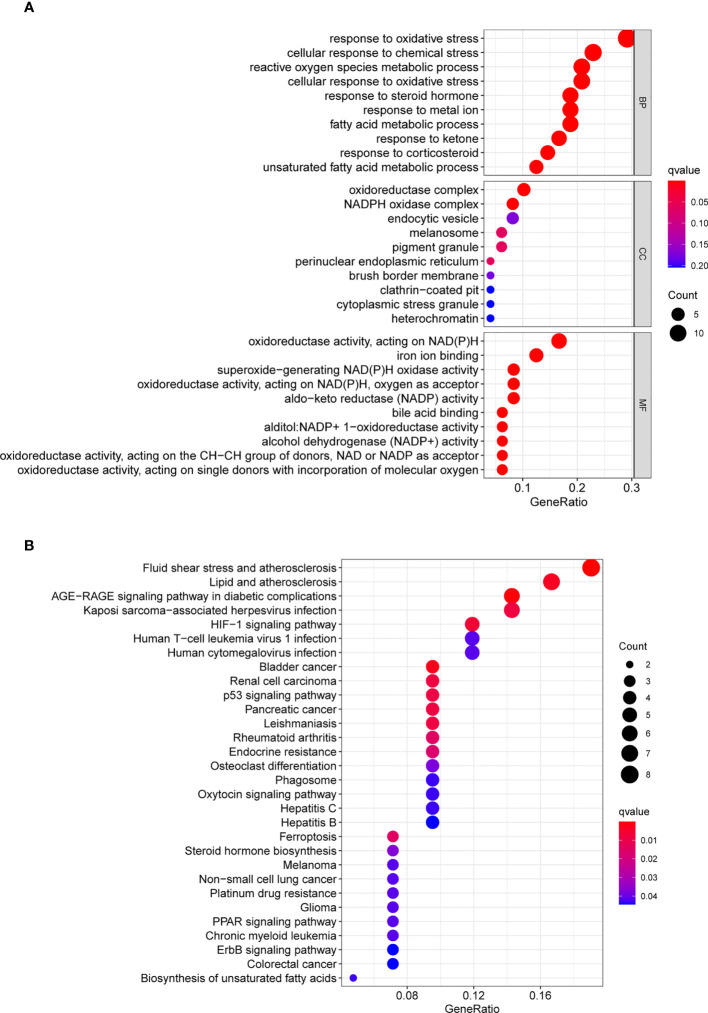
GO and KEGG analyses were applied to explore the potential function of ferroptosis-related differentially expressed genes. **(A)** GO assays and **(B)** KEGG assays.

Firstly, we screened ferroptosis-related DEGs between GC specimens and non-tumor specimens on the basis of TGCA datasets, and identified 50 ferroptosis-related DEGs, including 20 down-regulated ferroptosis-related DEGs and 30 upregulated ferroptosis-related DEGs. Then, we used these genes to fulfill GO and KEGG assays. We found that the 50 ferroptosis-related DEGs were mainly associated with reaction to oxidative stress, cellular reaction to chemical stress, reactive oxygen species metabolic process, oxidoreductase complex, NADPH oxidase complex, endocytic vesicle, oxidoreductase activity, acting on NAD(P)H, iron ion binding and superoxide-generating NAD(P)H oxidase activity (**Figure 1A**). KEGG assays revealed that the 50 ferroptosis-related DEGs were mainly concentrated in Fluid shear stress and atherosclerosis, Lipid and atherosclerosis, AGE-RAGE signaling pathway in diabetic complications, Kaposi sarcoma-associated herpesvirus infection, HIF-1 signaling pathway, Human T-cell leukemia virus 1 infection and Human cytomegalovirus infection (**Figure 1B**).

### Identification of the survival-related FRlncRNAs

**Figure 2:**
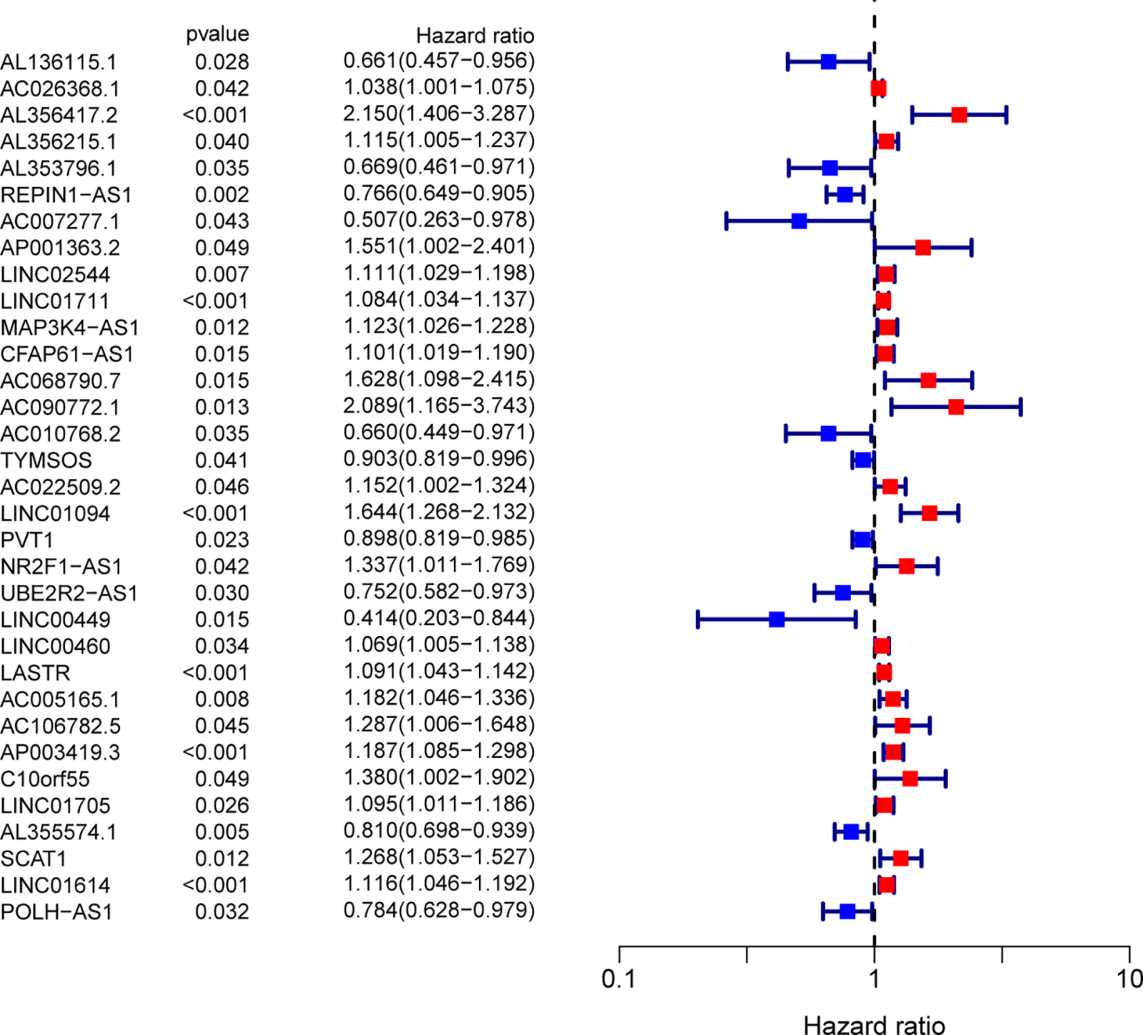
Ferroptosis-related lncRNAs related to survival were assessed by means of univariate Cox regression analysis.

We uncovered 1336 ferroptosis-related lncRNAs. To identify the survival-related FRlncRNAs, Univariate Cox regression analysis was conducted and 33 survival-related FRlncRNAs were identified (**Figure 2**). Among the above lncRNAs, it has been reported that some of them participant in the progression of several tumors, such as GC.

### Identification of the dysregulated survival-related FRlcnRNAs in our cohort

**Figure 3:**
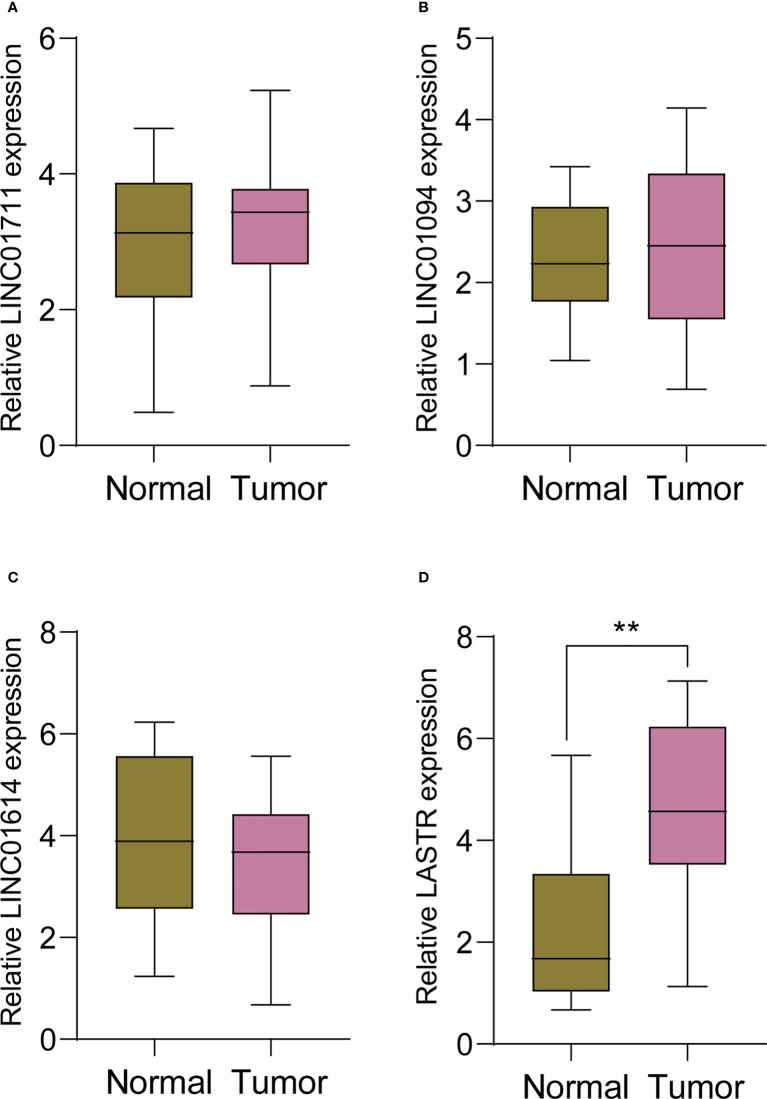
RT-PCR was applied to examine the expression of **(A)** LINC0711, **(B)** LINC01094, **(C)** LINC01614 and **(D)** LASTR in 11 pairs of GC specimens and matched non-tumor specimens. **p<0.01.

In order to screen critical FRlcnRNAs, we performed RT-PCR using four survival-related FRlncRNAs, including LINC0711, LINC01094, LINC01614 and LASTR. We found that the expression of LINC0711, LINC01094 and LINC01614 failed to exhibit an apparent difference between GC samples and non-tumor samples(**Figures 3A-C**). However, LASTR expressions were evidently increased in GC specimens (**Figure 3D**). Then, our attention focused on LASTR.

### The expression of LASTR in GC and its association with clinical factors based on TCGA datasets

**Figure 4:**
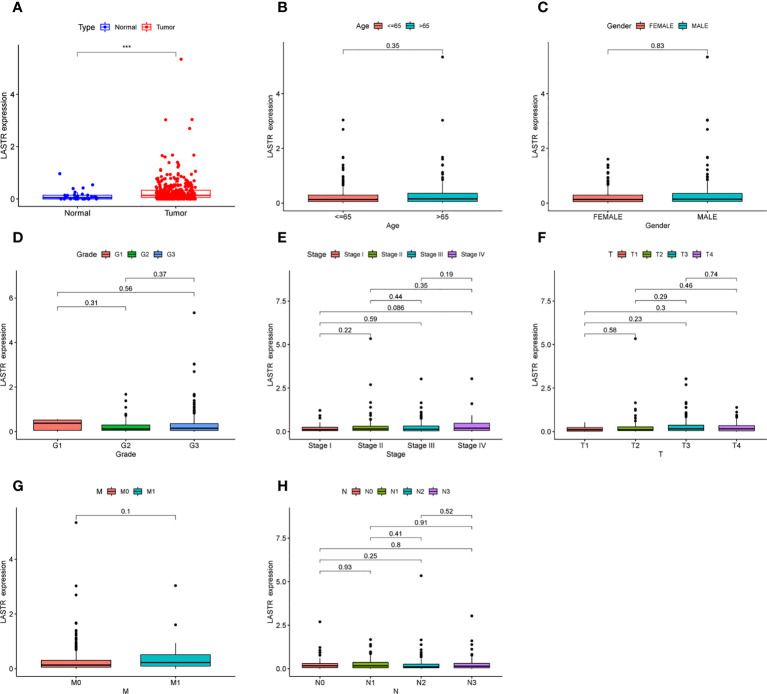
The expression of LASTR in specimens and its clinical significance. **(A)** LASTR expression was distinctly upregulated in GC specimens based on TCGA datasets. **(B-H)** The correlation between LASTR and clinicopathological characteristics. ***p<0.001.

Then, to further identify LASTR’s expression in GC, TCGA datasets were analyzed. Consequently, it was found that the expression of LASTR was distinctly increased in GC specimens compared to non-tumor specimens (**Figure 4A**). Moreover, efforts were made to study the relationship between LASTR expressions and clinical factors in GC patients. However, it was found that LASTR expression was not associated with age (**Figure 4B**), gender (**Figure 4C**), grade (**Figure 4D**), clinical stage (**Figure 4E**), T stage (**Figure 4F**), M stage (**Figure 4G**) and N stage (**Figure 4H**).

### The prognostic value of LASTR expression in GC patients

**Figure 5:**
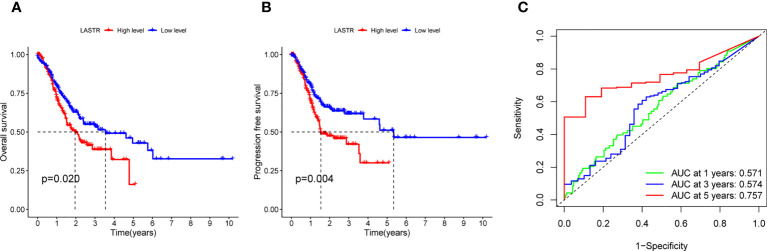
Prognostic value of LASTR among GC patients. **(A, B)** Kaplan-Meier plots of OS and PFS among GC patients with low and high level of LASTR. **(C)** ROC curves for the model representing 1-, 3-, and 5-year predictions. The values in brackets are the areas under the curve.

**Figure 6:**
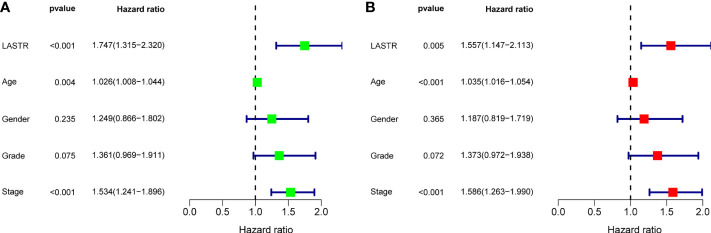
Univariate **(A)** and multivariate **(B)** analyses of overall survival among GC patients.

A Kaplan-Meier survival analysis and log-rank tests were employed. The results revealed that patients in high LASTR expression group showed poorer OS(p=0.020, **Figure 5A**) and PFS(p=0.004, **Figure 5B**) compared with those in low LASTR expression group (p = 0.0010). Moreover, the time-dependent ROC curve was conducted for the estimation of the performance of the risk prediction model. The AUC of LASTR was 0.571, 0.574, and 0.757 at 1, 3, and 5 years, respectively (**Figure 5C**). To further examine the clinical value of LASTR expressions in GC patients, univariate and multivariate assays were carried out based on Cox’s proportional hazard model. According to the univariate analysis, age, clinical stage, and LASTR expression were greatly linked to the overall survival of GC patients (**Figure 6A**). Moreover, through the multivariate Cox regression analyses, it was confirmed that the LASTR expression was an independent prognostic indicator to the overall survival (HR = 1.557; 95% CI, 1.147-2.113; p = 0.005) among GC patients (**Figure 6B**). These outcomes showed that LASTR expression might be consider as an important independent prognostic factor.

### Knockdown of LASTR expression suppressed the proliferation of GC cells

**Figure 7:**
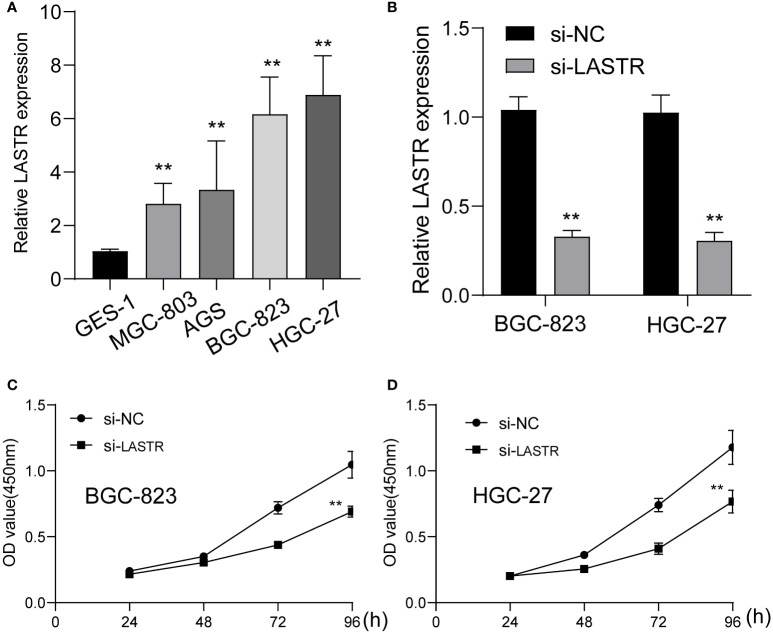
The function of LASTR silence on the proliferation of GC cells. **(A)** The expressions of LASTR were confirmed in GES-1 and four GC cell lines GES-1, HGC-27, MGC-803 and BGC-823. **(B)** Stable knockdown of LASTR was checked by RT-PCR in MGC-823 and HGC-27 cells transfected with si-LASTR. **(C, D)** The effect of LASTR expression on cell proliferation was assessed using MGC-823 and HGC-27 cells by CCK-8 assays. **p<0.01.

To investigate whether LASTR knockdown inhibited GC cell growth, at first, the expression of LASTR in four GC cells was examined using RT-PCR, and it was found that LASTR expression was greatly increased in four GC cells compared to GES-1 (**Figure 7A**). Because LASTR exhibited a higher level in BGC-823 and HGC-27 cells, we chose them for further *in vitro* experiments. To probe the biological function of LASTR, we designed small interfering RNAs (siRNAs) that can specifically target LASTR. As shown in **Figure 7B**, LASTR was effectively silenced by siRNA. Then, we assessed whether the knockdown of LASTR could affect the biological function of BGC-823 and HGC-27. The outcomes of CCK-8 assay revealed that knockdown of LASTR greatly restrained the proliferation of BGC-823 and HGC-27 cells (**Figures 7C, D**).

### Association between LASTR expressions and immune infiltrating levels in GC

**Figure 8:**
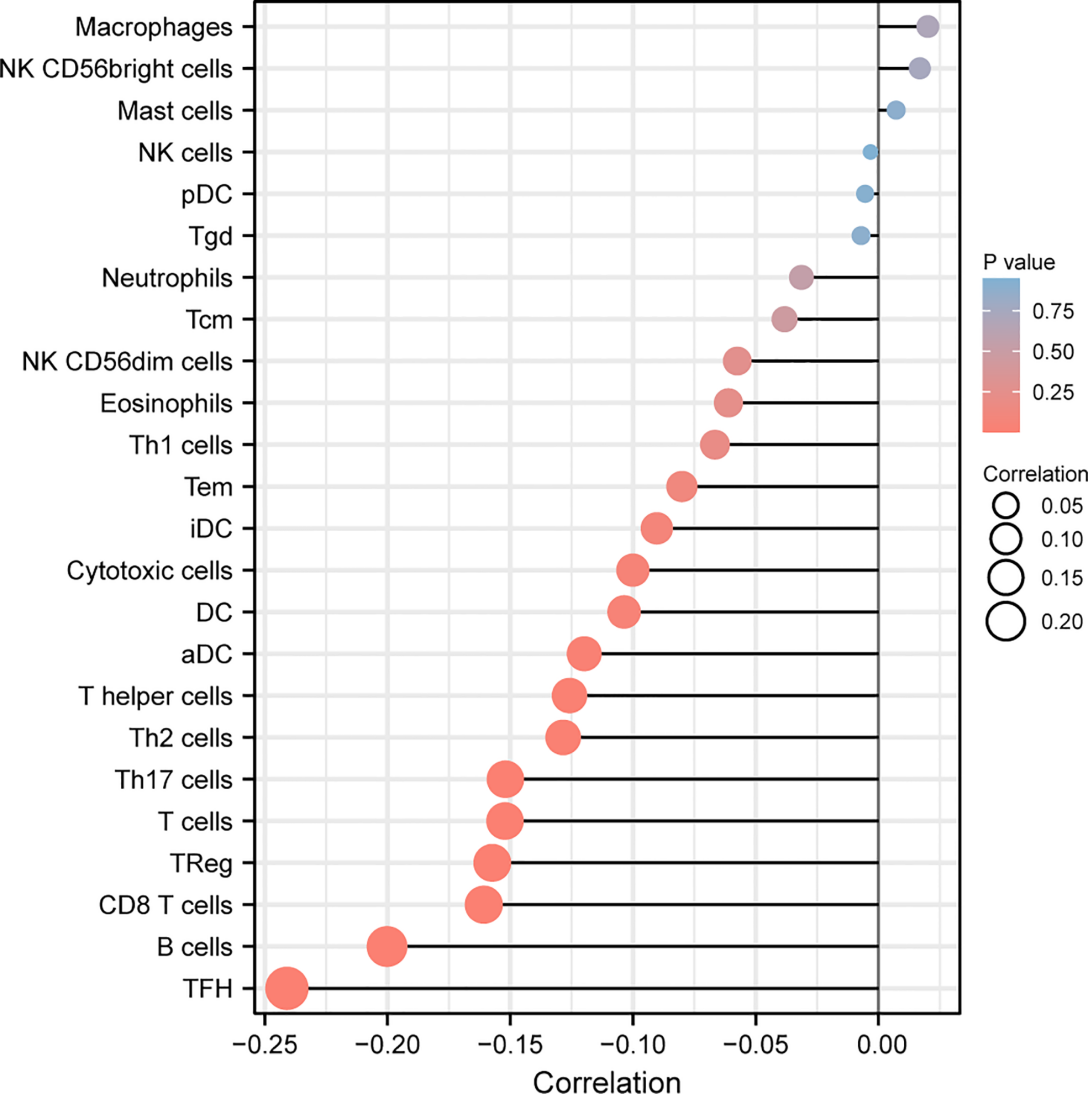
Correlation of LASTR expression with immune infiltration level in GC. The expression of LASTR was associated with several immune cells, such as TFH and B cells.

Then, we explored the correlation between immune infiltration and LASTR expression. As shown in **Figure 8**, we found that the expression of LASTR was negatively associated with TFH, B cells, CD8 T cells, Treg, T cells, Th17 cells and T helper cells.

## Discussion

Since GC is a highly heterogeneous malady featuring a high death rate and imperceptible symptoms, patients tend to be diagnosed with it until the late stage (28). Though the treatments for GC is updated continuously, the 5‐year survival rate remains disappointing. The first choice for better foreseeing tumor behavior and instructing the treatment scheme is to determine the informative diagnostic and prognostic biomarkers of GC (29, 30). So far, scientists have found that lncRNAs regulate target gene expression and acts as oncogenes or tumor suppressors. As the fast growth of high throughput genomic sequencing technologies, lncRNAs prove to be valuable biomarkers to more accurately assess the prognosis of various tumors.

Mounting research reveals a connection between autophagy and ferroptosis and tumor growth, as these two RCD subtypes are inextricably linked (22). Nevertheless, the involvement of lncRNAs in GC autophagic and ferroptotic processes was under-studied. This study screened ferroptosis-related DEGs between GC specimens and non-tumor specimens on the basis of TGCA datasets, and identified 50 ferroptosis-related DEGs, including 20 down-regulated ferroptosis-related DEGs and 30 upregulated ferroptosis-related DEGs. Furthermore, KEGG assays illuminated that the 50 ferroptosis-related DEGs were mainly concentrated in Fluid shear stress and atherosclerosis, Lipid and atherosclerosis, AGE-RAGE signaling pathway in diabetic complications, Kaposi sarcoma−associated herpesvirus infection, HIF-1 signaling pathway, Human T-cell leukemia virus 1 infection and Human cytomegalovirus infection, suggesting these ferroptosis-related DEGs play a central part in the development of tumors. Then, 33 survival-related FRlncRNAs were identified. We chose LINC0711, LINC01094, LINC01614 and LASTR to demonstrate their expression in GC. Interestingly, we only found that LASTR expression was significantly increased in GC specimens in this cohort, which was further confirmed in TCGA datasets. These findings supported that LASTR was an important player in GC.

In recent years, only a few studies mentioned the function of LASTR in some tumors. For example, it was reported that the expressions of LASTR in lung cancer samples (LUAD and LUSC) were significantly higher than those in neighboring normal tissue. Patients whose LASTR expression levels were higher had a shorter overall survival and worse clinical characteristics, as determined by the Kaplan-Meier survival analysis, in comparison to patients whose LASTR expression levels were lower. This resulted in a lower overall survival rate. Knockdown of LASTR considerably curbed the proliferation and metastatic ability of lung cancer cells through the miRNA-137/TGFA axis (31). After that, the further analysis of the prognostic value of LASTR expression in GC patients found that high LASTR expression was related to shorter OS and PFS in GC patients. More importantly, through multivariate Cox regression analysis, it was confirmed that the LASTR expression was an independent prognostic indicator to the overall survival of GC patients. Finally, the loss of function experiments was performed and it was observed that the knockdown of LASTR greatly restrain the proliferation of GC cells, indicating that it is an oncogenic lncRNA in GC progression.

The tumor microenvironment, which harbors multiple immune and stromal cell types, is a key determinant of tumor progression and antitumor immunity (32). Growing studies have reported the positive associations between lncRNAs and TME immune cell infiltration (33, 34). In our study, we also observed the expression of LASTR was negatively associated with TFH, B cells, CD8 T cells, Treg, T cells, Th17 cells and T helper cells. Our findings suggested that LASTR may be a potential biomarker for predicting response to cancer immunotherapy.

One limitation of this study was that since it is a retrospective study, missing data and selection biases were unavoidable. Another limitation was that, values of gene expressions obtained from RNA‐seqs or microarrays were all relative. Hence, it was impossible to determine the absolute thresholds of stratifications in different cohorts. With median cutoff values involved in each data, there will be a need for accurate external validations. In addition, we just performed *in vitro* experiments to test the function of LASTR knockdown on the proliferation of GC cells, more *in vivo* experiments were needed to further confirm our findings.

## Conclusion

We found LASTR as an overexpressed lncRNA in GC. Meanwhile, LASTR was validated to be a novel prognostic biomarker for patients with GC. Moreover, knockdown of LASTR was confirmed to inhibit the proliferation of GC cells. The facts above pointed out that LASTR might be quite vital for the diagnosis and development of GC, and could even become an important therapeutic target for GC patients.

## Data availability statement

The original contributions presented in the study are included in the article/supplementary materials. Further inquiries can be directed to the corresponding authors.

## Ethics statement

The studies involving human participants were reviewed and approved by The First Medical Center, Chinese PLA General Hospital. The patients/participants provided their written informed consent to participate in this study.

## Author contributions

J-YL, JY, J-XC and X-DY designed the research. J-YL, J-JL, TH and F-JW performed the research and collected the data. JY, F-JW and T-YX analyzed the data. J-Y: and JY drafted the manuscript. All authors contributed to the article and approved the submitted version.

## Funding

This work was supported by the State Key Program of National Natural Science Foundation of China(No. NO.62133010).

## Conflict of interest

The authors declare that the research was conducted in the absence of any commercial or financial relationships that could be construed as a potential conflict of interest.

## Publisher’s note

All claims expressed in this article are solely those of the authors and do not necessarily represent those of their affiliated organizations, or those of the publisher, the editors and the reviewers. Any product that may be evaluated in this article, or claim that may be made by its manufacturer, is not guaranteed or endorsed by the publisher.
